# Digital quantification of p16-positive foci in fibrotic interstitial lung disease is associated with a phenotype of idiopathic pulmonary fibrosis with reduced survival

**DOI:** 10.1186/s12931-022-02067-w

**Published:** 2022-06-07

**Authors:** Jonathan Keow, Matthew J. Cecchini, Nathashi Jayawardena, Maurizio Zompatori, Mariamma G. Joseph, Marco Mura

**Affiliations:** 1 Department of Pathology and Laboratory Medicine, Western University, London, ON Canada; 2 Interstitial Lung Disease Research Laboratory, Lawson Research Institute, Western University, London, ON Canada; 3 MultiMedica Group, I.R.C.C.S. San Giuseppe Hospital, Milan, Italy; 4 Division of Respirology, Department of Medicine, Western University, London, ON Canada

**Keywords:** Senescence, Interstitial lung disease, Idiopathic pulmonary fibrosis, Usual interstitial pneumonia

## Abstract

**Background:**

Idiopathic pulmonary fibrosis (IPF) is associated with increased expression of cyclin-dependent kinase inhibitors such as p16 and p21, and subsequent induction of cell cycle arrest, cellular senescence, and pro-fibrotic gene expression. We sought to link p16-expression with a diagnosis of IPF or other fibrotic interstitial lung diseases (ILDs), radiographic pattern, senescent foci-specific gene expression, antifibrotic therapy response, and lung transplant (LTx)-free survival.

**Methods:**

Eighty-six cases of fibrosing ILD were identified with surgical lung biopsy. Immunohistochemistry for p16 was performed on sections with the most active fibrosis. p16-positive foci (loose collection of p16-positive fibroblasts with overlying p16-positive epithelium) were identified on digital slides and quantified. Cases were scored as p16-low (≤ 2.1 foci per 100 mm^2^) or p16-high (> 2.1 foci per 100 mm^2^). Twenty-four areas including senescent foci, fibrotic and normal areas were characterized using in situ RNA expression analysis with digital spatial profiling (DSP) in selected cases.

**Results:**

The presence of p16-positive foci was specific for the diagnosis of IPF, where 50% of cases expressed any level of p16 and 26% were p16-high. There was no relationship between radiographic pattern and p16 expression. However, there was increased expression of cyclin-dependent kinase inhibitors, collagens and matrix remodeling genes within p16-positive foci, and cases with high p16 expression had shorter LTx-free survival. On the other hand, antifibrotic therapy was significantly protective. DSP demonstrated that fibroblastic foci exhibit transcriptional features clearly distinct from that of normal-looking and even fibrotic areas.

**Conclusions:**

We demonstrated the potential clinical applicability of a standardized quantification of p16-positive fibroblastic foci. This method identifies an IPF phenotype associated with foci-specific upregulation of senescence-associated and matrix remodeling gene expression. While these patients have reduced LTx-free survival, good response to antifibrotic therapies was observed in those who were treated.

**Supplementary Information:**

The online version contains supplementary material available at 10.1186/s12931-022-02067-w.

## Background

Interstitial lung disease (ILD) encompasses a spectrum of conditions characterized by inflammation and fibrosis of the pulmonary interstitium. Although there are many forms of fibrotic ILD, the most common pathologic patterns are usual interstitial pneumonia (UIP) and nonspecific interstitial pneumonia (NSIP). Many cases of fibrotic ILD are diagnosed solely based upon their imaging characteristics [1], but there are a subset of cases that require surgical lung biopsy (SLB) for definitive characterization of the underlying ILD. Accurate classification is essential due to the significant difference in the treatment and prognosis of different forms of ILD. However, there are often overlapping features of different types of fibrotic ILD, making these cases challenging [2].

Many cases of UIP can be clinically classified as idiopathic pulmonary fibrosis (IPF) in the absence of any other known cause of a UIP pattern of injury (including autoimmune diseases). IPF carries a poor prognosis in the absence of therapy, with a median survival of 3–5 years from time of diagnosis [3–5]. Antifibrotic agents have some limited benefit in delaying the progression of IPF [6, 7], but the only definitive therapy of IPF is lung transplant (LTx) [8].

Histologically, UIP is characterized by a patchy subpleural fibrosis that is temporally and spatially heterogeneous. Areas of older, dense fibrosis with microscopic honeycombing are intermixed with areas of active fibrosis characterized by fibroblastic foci and uninvolved lung parenchyma. UIP patterns predict a worse prognosis [9, 10] when compared to other patterns on SLB. However, conflicting data have been published regarding the hypothesis that other morphological findings, such as the extent of traction bronchiectasis [11] or density of fibroblastic foci [12, 13], can predict outcome. The role of fibroblastic foci in the progression of pulmonary fibrosis may instead depend on their activity and current pathobiological role in each individual case. Therefore, none of these findings appears consistently predictive for prognostic outcome and are not routinely assessed in clinical practice.

Abnormal cellular senescence has been identified as a keystone process in the development of pulmonary fibrosis [14–16], and a subset of fibrotic ILD has been linked to abnormal cellular senescence. A family of cyclin-dependent kinase (CDK) inhibitors (including p15, p16 and p21) initiate cell cycle arrest in response to context or cell type specific triggers, such as DNA damage and other cellular stressors [17]. The induction of cellular senescence in these cases of pulmonary fibrosis may be mediated by p16 activation [18, 19], arresting the cell cycle through activation of this retinoblastoma-mediated CDK 4/6 pathway. Although this growing body of evidence supports the role of senescence in driving the development of pulmonary fibrosis, the specificity of senescence for IPF and relevance of senescence across the spectrum of fibrotic ILD are both unknown. None of these studies have yet provided a means to quantify these foci in a clinically deployable test, using standardized methodology.

Assays assessing for p16 expression are commonly used in many clinical laboratories, serving as a marker of high-grade squamous lesions associated with human Papilloma virus infection. These well-developed immunohistochemical tools can detect p16 expression, but are unavailable for many of the other CDK inhibitors. By leveraging these assays for p16, we developed a reproducible and clinically deployable method for identifying and quantifying senescent foci defined by p16 expression in epithelial cells and their underlying myofibroblasts. We therefore sought to apply this method on SLBs to identify a UIP pattern, and demonstrate a “pro-senescent” phenotype potentially associated with distinct HRCT pattern, RNA expression profile, prognosis, and response to therapy. Specifically, digital spatial RNA high-resolution profiling with the GeoMX platform was used for the first time in fibrotic ILD to explore transcriptional differences in fibroblastic foci vs. fibrotic and normal-looking areas in IPF cases.

## Materials and methods

### Case selection

**Fig. 1:**
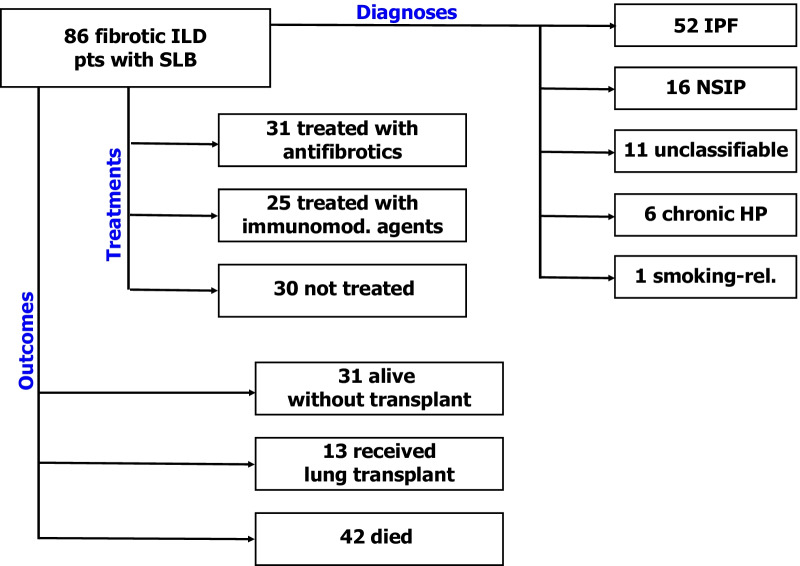
Diagram illustrating patients’ diagnoses, treatments and outcomes

Eighty-six cases of ILD diagnosed with SLB were identified between 2003 and 2018 at a tertiary-care level hospital (Table 1, Fig. 1). The general diagnosis of fibrotic ILD was first determined by chart review. After excluding all known causes of ILD (including autoimmune disease) and in the absence of a typical UIP radiographic pattern on high resolution CT scan (HRCT), patients were referred to SLB to clarify the specific type of fibrotic ILD. The final diagnosis of each case of fibrotic ILD was based on clinical-radiographic-pathologic multi-disciplinary discussion (MDD) [20].

**Table 1:** Patients’ characteristics at the time of surgical lung biopsy and outcomes

Diagnosis	n (%)
IPF	52 (60)
NSIP	16 (19)
Unclassifiable	11 (13)
Chronic HP	6 (7)
Smoking-related	1 (1)

Demographics/lung function	Mean ± SD
Age (years)	62 ± 10
Male / Female (% male)	48 / 38 (56%)
BMI (kg/m.^2^)	30 ± 6
Pack-years	24 ± 19
FVC (% predicted)	75 ± 19
TLC (% predicted)	70 ± 15
DLCO (% predicted)	48 ± 17

Radiographic patterns	(%)
Probable UIP	37 (43)
Indeterminate for UIP	29 (34)
Inconsistent with UIP	20 (23)
HRCT visual fibrosis score	20 ± 8

Outcomes	Mean ± SD
Post-biopsy follow-up	34 ± 35
Alive / transplanted / died	31 / 13 / 42

*IPF* idiopathic pulmonary fibrosis, *NSIP* nonspecific interstitial pneumonia, *HP* hypersensitivity pneumonitis, *BMI* body mass index, *FVC* forced vital capacity, *TLC* total lung capacity. *DLCO* diffusion lung capacity for carbon monoxide, *UIP* usual interstitial pneumonia

All patient characteristics were collected at time of biopsy. Pulmonary function tests (PFTs) were performed according to European Respiratory Society and American Thoracic Society guidelines [21–24]. The HRCT pattern was evaluated and classified by a chest radiologist (MZ) who was blinded from clinical information, and classified as either probable UIP, indeterminate for UIP or inconsistent for UIP [25]. The overall extent of pulmonary fibrosis, including reticular, ground glass opacities and honeycomb changes, was then evaluated with a quantitative visual score at 4 pre-defined levels (aortic arch, bronchus intermedius, pulmonary veins, lowest scan) in each hemithorax. A 5-point scale representing extent of disease (0 = absence of lesions, 1, 2, 3, 4 = extent of lesions, respectively, 0–25%, 25–50%, 50–75%, 75–100%) was used for the score in each location. The component scores for each scan were then summed, creating a final overall score (100/maximum predicted value, equivalent to 8 times the number of scans performed). The extent of fibrosis was then expressed as a percentage of the total lung volume [26]. Subjects with incomplete follow-up data were excluded.

### Immunohistochemical analysis

Formalin-fixed paraffin-embedded tissue sampled from the SLB for each patient was used for immunohistochemical analysis. Haematoxylin and eosin (H&E) stained slides from each case were re-examined and reviewed by a pulmonary pathologist (MJC) blinded from clinical information. Sections with the greatest number of fibroblastic foci or the most active fibrosis were selected. Four micrometer-thick sections were then cut from these tissues for p16 (monoclonal antibody E6H4, Roche, Basel, Switzerland), p21 (monoclonal antibody EPR362, Abcam, Cambridge, United kingdom) and purified retinoblastoma protein (pRB)(monoclonal antibody G3-245, Biosciences, Mississauga, Canada) immunohistochemistry.

### Digital quantification

**Fig. 2:**
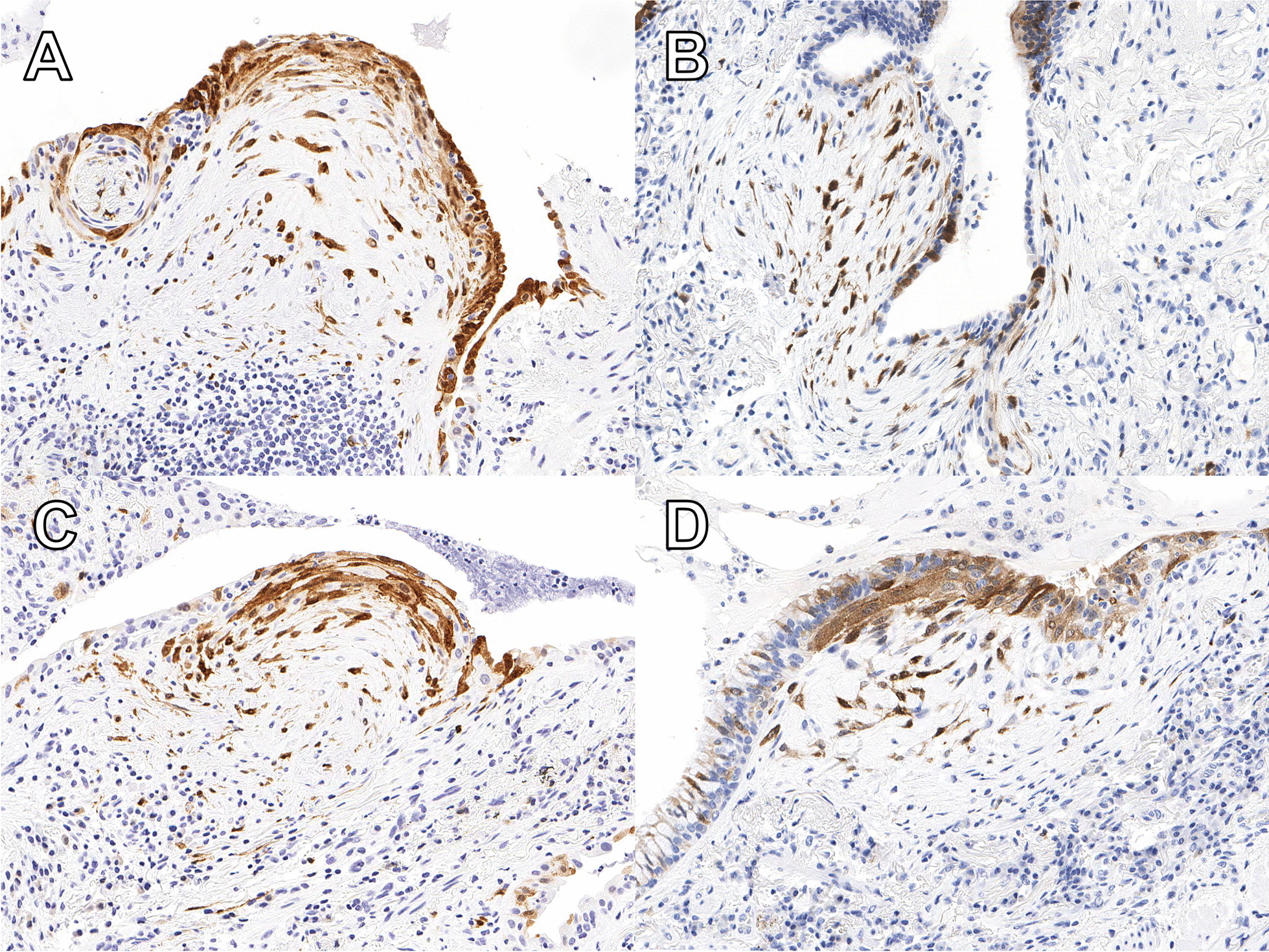
p16-positive foci were defined as concurrent expression of p16 (brown) in loose collections of fibroblasts the overlying flat (**A**), simple cuboidal (**B** and **C**) or columnar epithelium (**D**). Cases classified as p16-low had ≤ 2.1 foci per 100 mm^2^ lung tissue, and cases classified as p16-high had > 2.1 foci per 100 mm^2^ lung tissue

The immunohistochemical-stained slides and corresponding H&E slides were digitized using an Aperio ScanScope AT Turbo (Leica Biosystems, Wetzlar, Germany). Using these digital slides, p16-positive foci were defined as loose collections of p16-positive fibroblasts with an overlying p16-positive overlying flat (Fig. 2A), simple cuboidal (Fig. 2B and C) or columnar epithelium (Fig. 2D). Other areas of p16-positivity were disregarded. The total area of lung tissue and location of each p16-positive focus from each case was annotated on the digital slide (Additional file 2: Fig. S1) using the open access QuPath software package [27]. The total surface area was then calculated based on the annotated areas in a semi-automated protocol. The number of p16-positive foci for each case was normalized/100 mm^2^ lung tissue, and cases were scored as p16-low (≤ 2.1 foci per 100 mm^2^ lung tissue) or p16-high (> 2.1 foci per 100 mm^2^ lung tissue), based on receiver operating characteristics analysis (c-statistics) against survival.

### Digital spatial RNA profiling

The GeoMX Digital Spatial Profiling (DSP)(NanoString Technologies, Seattle, WA) is a method providing highly multiplex spatial profiling of RNAs on formalin-fixed, paraffin-embedded (FFPE) samples [28]. The DSP platform quantifies the abundance of RNA by counting unique indexing oligonucleotides assigned to each target of interest. The GeoMX DSP allows for up to 800-plex profiling of mRNA or protein using an optical-barcode readout [28]. Slide-mounted FFPE tissue sections underwent antigen retrieval and incubation with indexing oligonucleotides covalently attached to mRNA hybridization probes with a UV-photocleavable (PC) linker [28]. The same sections were stained with fluorescently labeled imaging reagents to identify tissue features and markers of interest. The system is highly sensitive and non-destructive, allowing multiple cycles of high-plex profiling on the same tissue section or subsequent DNA sequencing on the same section.

**Fig. 3:**
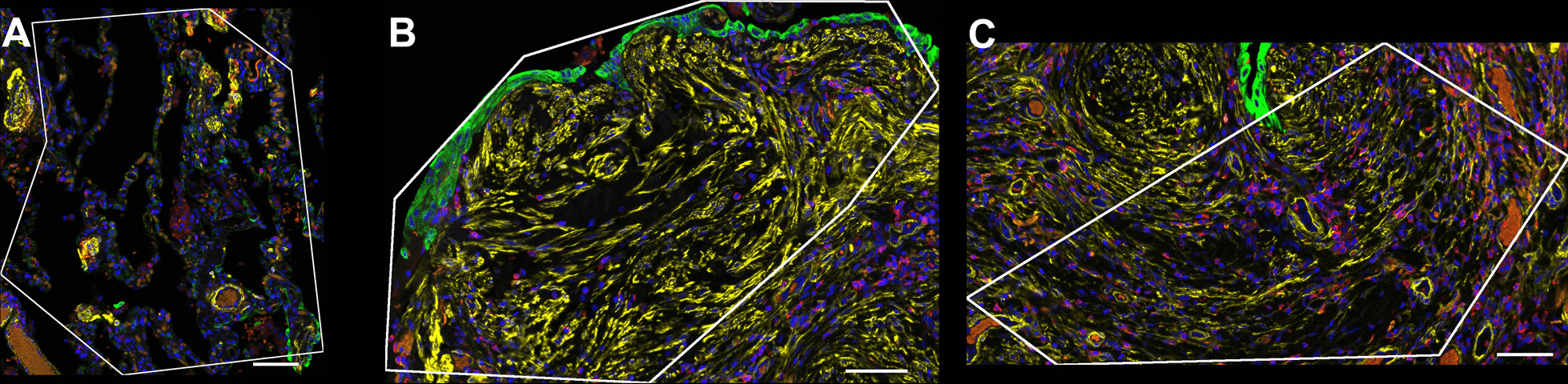
Regions of Interest (ROI) were selected using immunofluorescence markers for cytokeratin (green), smooth muscle actin (yellow), CD45 (red), and DAPI (blue) to highlight the different cellular and extracellular characteristics of each area of interest. Representative areas of normal lung (**A**), fibroblastic foci (**B**) and areas of dense fibrosis (**C**) were selected from 2 cases with a high density of p16-positive foci. Scale bar = 100 µm

We selected 24 lung microenvironments from FFPE sections in 2 cases of IPF, both with a high density of p16-positive foci. Tissue sections were stained with standard morphology markers (pan-cytokeratin, alpha-smooth muscle actin, CD45) combined with oligo-tagged ISH probes (RNA) that contained a photocleavable linker. Regions of interest (ROIs) were selected, including 12 fibroblastic foci, 6 areas of pulmonary fibrosis and 6 areas of normal lung tissue in total (Fig. 3). Areas of illumination were consequently identified by morphology markers, then sequentially exposed to UV light to decouple the oligonucleotides from the profiling reagents. Decoupled oligonucleotides were rapidly aspirated using a microcapillary without touching the sample, thereby leaving the sample unaltered. Oligonucleotides were deposited into wells of a microtiter plate, and the information contained within each well were indexed to the ROI on the tissue. The oligonucleotides were hybridized to NanoString® barcodes and sequenced using standard next generation sequencing technologies.

For quality control purposes, counts from each ROI were analyzed mRNAs were targeted by multiple probes. Outlier probes and under-sequenced samples were dropped from downstream data analysis. RNA was detected in a reproducible manner with high correlation to fluorescent in situ hybridization. Semi-quantitative analysis [29] of these ROIs was then performed by standard next generation oligonucleotide sequencing. In terms of normalization for reproducibility purposes, individual counts were normalized against the signal detected from 31 housekeeping genes across the study. Individual counts were also be normalized against the 75th percentile of signal from their own ROI.

The Cancer Transcriptome Atlas Assay (NanoString®) was used, as considered the most inclusive of genes of interest for pulmonary fibrosis. This atlas included 1825 genes, and principal component analysis mapping was carried out with the GeoMX software (NanoString®). Differentially expressed genes were selected based on both a q-value < 0.05 and a fold change of ≥ 1.5. For hierarchic clustering, Cluster 3.0 and Treeview (Eisen’s Laboratory, Stanford University, Stanford, CA) were used (http://rana.lbl.gov/eisen/?page_id=7). Unsupervised clustering does not take any of the experimental variables such as treatment, phenotype, tissue, etc. into account. For Gene-set Enrichment Analysis (GSEA), C5 GeneOntology was used as gene-set database [30].

### Statistical analysis

Pearson’s correlation was used to compare p16 expression and HRCT score. The Kolmogorov–Smirnov test assessed variables’ distribution. The Student's t-test, the χ^2^ method, and Fisher's exact test were used, when appropriate, to compare continuous and categorical variables. Univariate and multivariate Cox proportional hazards regression analyses were used to identify the significant predictors of LTx-free survival. Receiver operating characteristic (ROC) analysis (c-statistics) was used to determine best cut-off of p16 expression to predict LTx-free survival, by examining accuracy (sum of sensitivity and specificity). LTx-free survival from the time of biopsy was also evaluated using Kaplan–Meier curves and the log-rank test. p-values < 0.05 were regarded as significant. For RNA sequencing data, the q-value was adopted as a multiple comparison correction and used to identify differentially expressed genes. The q-value represents the minimal false discovery ratio (FDR) at which an individual hypothesis test may be called significant. Prism-8 (GraphPad, La Jolla, CA), JMP (SAS Institute, Cary, NC) and MedCalc (Mariakerke, Belgium) softwares were used.

## Results

### Patients characteristics and mortality

After SLB and MDD, 52 cases were diagnosed with IPF and 34 with fibrotic ILDs other than IPF (Table 1, Fig. 1). The average age of patients was 62 years, with a male predominance (56%). On average, these patients had a mild restrictive ventilatory defect (FVC 75%, TLC 70%), with moderate reduction of diffusing lung capacity (48%). HRCT visual score analysis demonstrated an average pre-biopsy extent of fibrosis of 20%.

Post-biopsy follow-up was 34 ± 35 months. Thirty-one patients (36%) were treated with antifibrotic therapy (either pirfenidone or nintedanib), 25 (29%) with immunomodulatory therapy (either prednisone, mycophenolate mofetil, azathioprine or cyclophosphamide) and 30 (35%) did not receive specific treatment (Fig. 1). Of note, several patients were diagnosed with IPF before the approval of pirfenidone (2013) and nintedanib (2015) in Canada. During the post-biopsy observation period, mortality was 49% and 13 patients (15%) underwent LTx (Fig. 1). The combined incidence of death and LTx was therefore 64%.

### p16-positive foci specificity to IPF

Positive p16 immunohistochemistry was consistently observed on the epithelium overlying numerous, but not all, fibroblastic foci in IPF cases (Fig. 2).

Any number of p16-positive foci were identified in 40 of the 52 cases of IPF. The presence of any p16-positive foci was sensitive (76.9%) and specific (91.2%) for the final diagnosis of IPF against other types of ILD. There was however a variable expression of p16 in the IPF group, with a range of p16-positive foci between 0 and 33 per slide (median: 3 foci per slide), and the density of senescent foci ranged from 0.43 to 26.32 foci per 100 mm^2^ lung tissue, with a median density of 1.97 foci per 100 mm^2^ lung tissue. We selected a cut-off of 2.1 foci per 100 mm^2^ to maximize the differences in outcome between the two groups, in accordance with the ROC analysis (Additional file 3: Fig. S2)*.* As a result, 19 cases of IPF (47.5%) were classified as p16-low (≤ 2.1 foci per 100 mm^2^ lung tissue), and 21 cases of IPF (52.5%) were scored as p16-high (> 2.1 foci per 100 mm^2^ lung tissue) (Table 2).

**Table 2:** Histopathologic characteristics of fibrosing ILD and associated p16 expression

Diagnosis	p16-low (%)	p16-high (%)
IPF	31 (60)	21 (40)
NSIP	16 (100)	0 (0)
Unclassifiable	10 (91)	1 (9)
Chronic HP	6 (100)	0 (0)
Smoking-related	1 (100)	0 (0)

Cases were subclassified into p16-low (≤ 2.1 foci per 100 mm^2^ of lung tissue) and p16-high (> 2.1 foci per 100 mm^2^ of lung tissue)

*IPF* idiopathic pulmonary fibrosis, *NSIP* nonspecific interstitial pneumonia, *HP* hypersensitivity pneumonitis

p16-positive foci were present in only 3 cases of non-IPF fibrotic ILD. Two of the cases had unclassifiable patterns of fibrotic ILD, with 3.82 and 1.08 foci per 100 mm^2^ lung tissue, respectively, and the remaining one was a case of chronic hypersensitivity pneumonitis with 1 p16-positive focus (1.32 foci per 100 mm^2^ lung tissue). Senescent foci were absent from all other cases of non-IPF, including 16 cases of NSIP, 5 cases of chronic hypersensitivity pneumonitis, and 11 unclassifiable cases. The specificity of p16-high for the diagnosis of IPF was 97.6%. No relationship was observed between the pre-biopsy HRCT pattern and the density of p16-positive foci (Additional file 4: Fig. S3). No significant associations were identified between PFTs or HRCT fibrosis score and the density of p16-positive foci (data not shown).

**Fig. 4:**
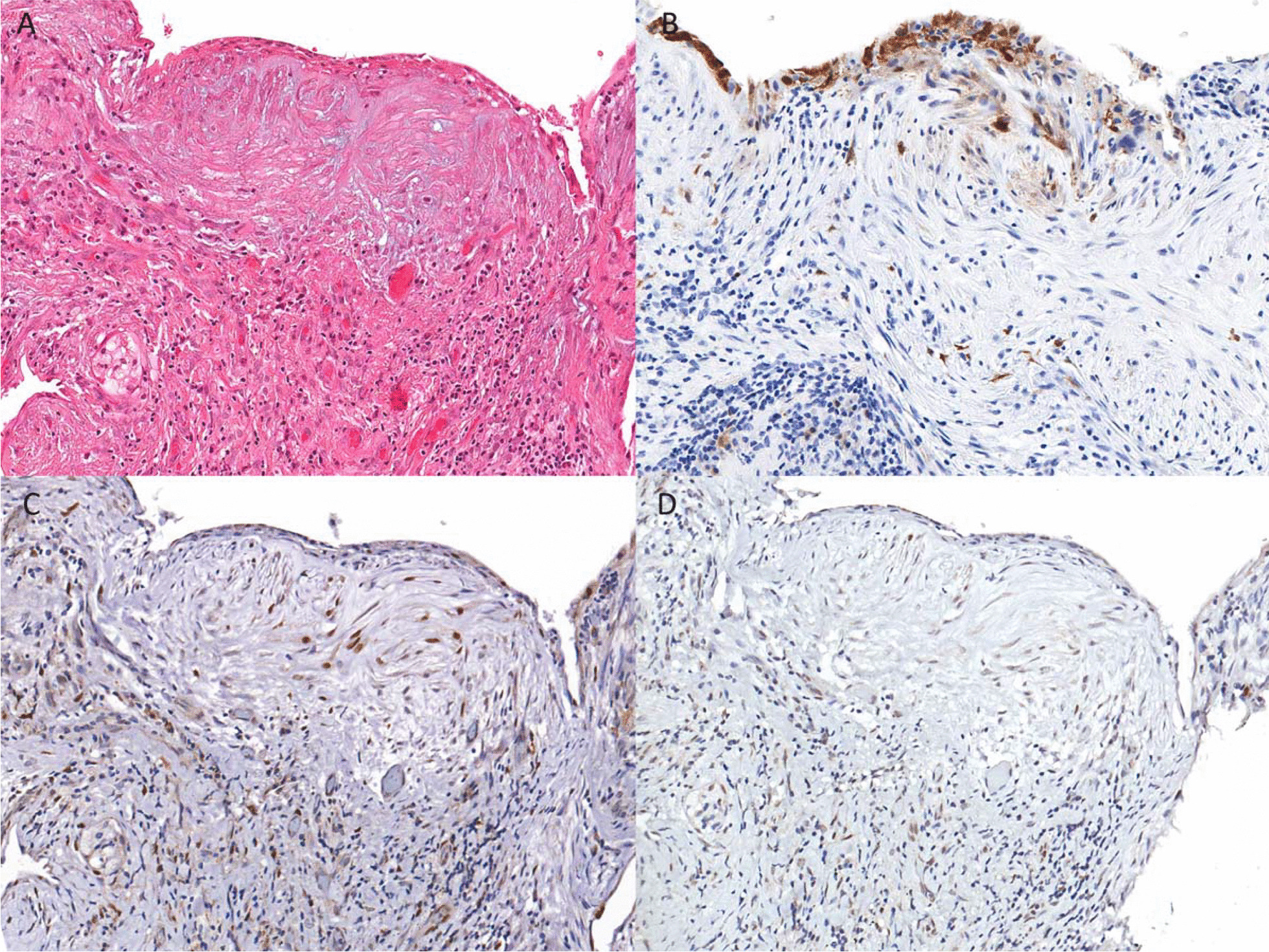
**A** Hematoxylin and eosin stained section of a fibroblastic focus in a case of IPF. **B** p16 immunohistochemistry of the same area. **C** p21 immunohistochemistry of the same area. **D** pRB immunohistochemistry of the same area. Objective magnification 20X

Additional markers of senescence were tested with immunohistochemistry. Material was available for 6 cases for p21 and pRB staining. These cases had a range of normalized p16 foci from 0 to 26.32. While the p16 positive foci were found to express pRB (Fig. 4) the expression was not substantially above the background staining seen in the lung and is in keeping with wild-type intact pRB expression. No significant accentuation in of pRB staining was present in the foci. As shown in Fig. 4, p21 is also expressed in p16 positive fibroblastic foci. It was not possible to directly compare all the p16 positive foci to the p21 staining due to variation in the tissue composition on deeper levels. Overall 85% of fibroblastic foci identified on histologic sections have some degree of p21 staining. However, the staining is focal, patchy and challenging to appreciate above the background patchy p21 staining.

### Digital spatial RNA profiling

**Fig. 5:**
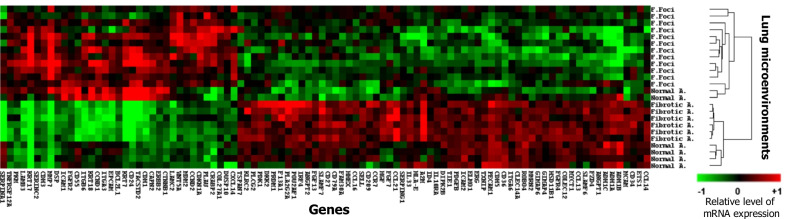
Heat map representing differentially expressed genes (fold change ≥ 1.5 and q value < 0.05) and hierarchic clustering based on 97 differentially expressed genes. Each row corresponds to an individual lung microenvironment, and each column corresponds to an individual gene. Each square on the matrix represents the expression level of an individual gene in each sample, with red and green indicating gene expression levels above or below, respectively, compared with each other. Relative levels of mRNA expression are illustrated with green (relative decrease, up to − 1), red (relative increase, up to + 1) or no change (black, 0). Unsupervised clustering analysis shows distinct homology of expression within the fibroblastic foci, divergent from the expression patterns within the areas of normal lung and dense fibrosis

**Fig. 6:**
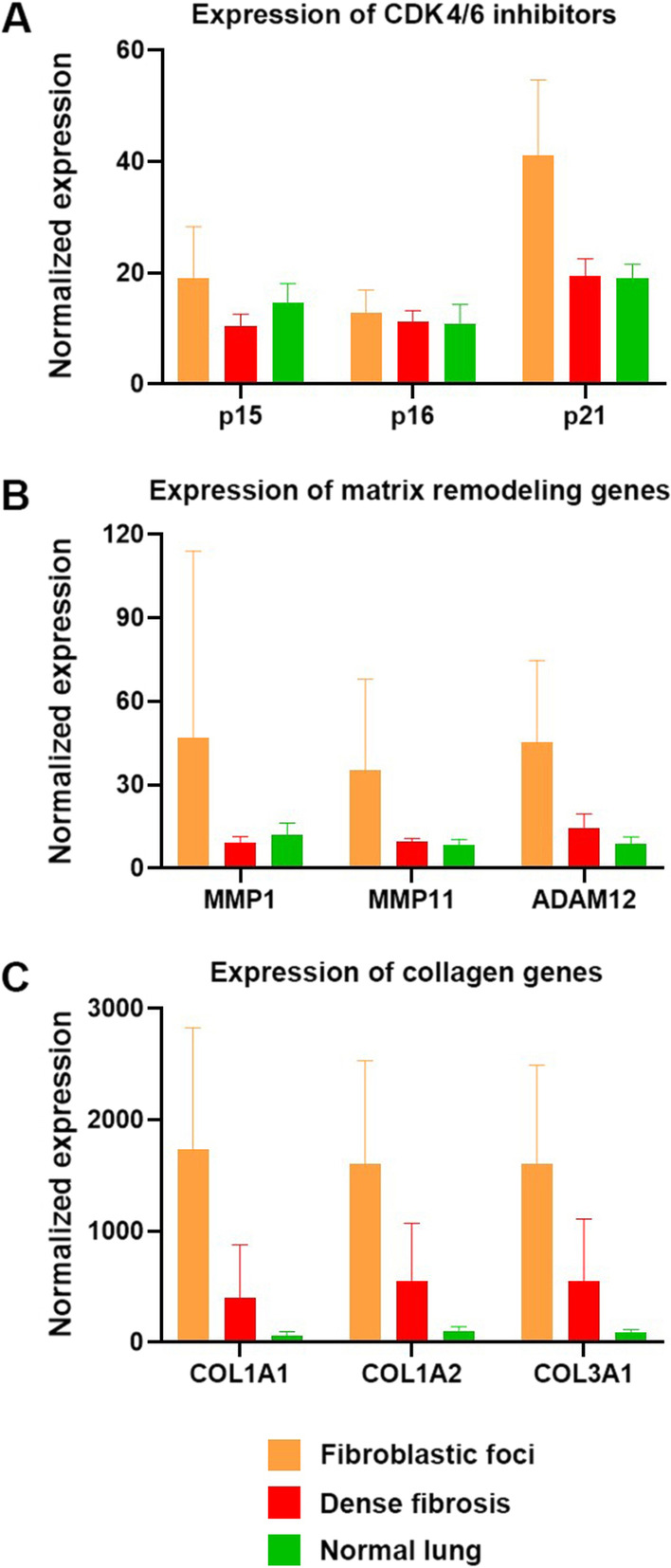
Normalized mRNA expression of CDK 4/ 6 inhibitors (**A**), matrix remodelling genes (**B**) and collagens (**C**) in fibroblastic foci (orange, n = 12), areas of dense fibrosis (red, n = 6), and areas of normal lung (green, n = 6) in tissue with a high density of p16-positive foci. Expression of p21, MMP1, MMP11, ADAM12, COL1A1, COL1A2, and COL3A1 are increased in fibroblastic foci when compared to areas of dense fibrosis and areas of normal lung

A compact and homogenous unsupervised clustering of fibroblastic foci gene expression was observed, separating them from both the fibrotic and normal areas (Fig. 5). Consistently, principal component analysis (PCA) mapping demonstrated a definite separation across the selected gene array among fibroblastic foci, fibrotic and normal areas (Additional file 5: Fig. S4). Applying strict criteria (q-value < 0.05, fold change ≥ 1.5), 97 differentially expressed genes were identified between the fibroblastic foci and zones of dense fibrosis or normal lung. RNA expression analysis revealed a distinct upregulation in CDK inhibitor p21, extracellular matrix remodelling proteases (MMP-1, MMP-11 and ADAM12), and collagens (Collagen 1A1, Collagen 1A2 and Collagen 3A1) in fibroblastic foci, compared to both areas of dense fibrosis and normal areas (Fig. 6).

GSEA demonstrated that 107 gene sets were upregulated in fibroblastic foci vs. normal-looking areas with an FDR < 0.05. Top 5 upregulated gene sets are shown in Table 3. Not surprisingly, “scarring” was the top upregulated gene set in fibroblastic foci. In contrast, only 4 gene sets were upregulated in fibrotic areas vs. normal-looking areas with an FDR < 0.05. Top 5 upregulated gene sets are shown in Table 4. The top upregulated gene set in fibrotic areas was related to the process of endocytosis. The comparison of fibroblastic foci with fibrotic areas yielded as many as 21 upregulated gene sets with an FDR < 0.05 in the first group (Table 5).

**Table 3:** Top 5 gene-sets significantly enriched in the fibroblastic foci vs. normal-looking areas (GSEA)

Gene Set	NES	p value	q value
Human phenotype scarring	2.47	0.000	0.000
Atrophic scars	2.39	0.000	0.000
Abnormality of limb epiphysis morphology	2.29	0.000	0.000
Dermal atrophy	2.26	0.000	0.001
Aplasia/Hypoplasia of the skin	2.25	0.000	0.001

The positive enrichment score indicated a correlation with the fibroblastic foci microenvironment group

*NES* normalized enrichment score

q value: false discovery ratio

**Table 4:** Top 5 gene-sets significantly enriched in the fibrotic areas vs normal-looking areas group (GSEA)

Gene Set	NES	p value	q value
Regulation of receptor-mediated endocytosis	2.07	0.000	0.028
Positive regulation of receptor-mediated endocytosis	2.03	0.000	0.035
Dilatation of the cerebral artery	2.02	0.000	0.025
Hyperextensible skin	2.01	0.000	0.023
Aortic aneurysm	1.95	0.000	0.062

The positive enrichment score indicated a correlation with the fibrotic areas microenvironment group

*NES* normalized enrichment score

q value: false discovery ratio

**Table 5:** Top 5 gene-sets significantly enriched in the fibroblastic foci vs. fibrotic areas (GSEA)

Gene Set	NES	p value	q value
Cadherin binding	2.40	0.000	0.012
Structural molecular activity	2.36	0.000	0.016
Extracellular matrix structural constituent conferring tensile strength	2.34	0.000	0.020
Abnormality of limb epiphysis morphology	2.29	0.000	0.034
Extracellular matrix structural constituent	2.27	0.000	0.046

The positive enrichment score indicated a correlation with the fibroblastic foci microenvironment group

*NES* normalized enrichment score

q value: false discovery ratio

### Post-biopsy survival analysis

**Fig. 7:**
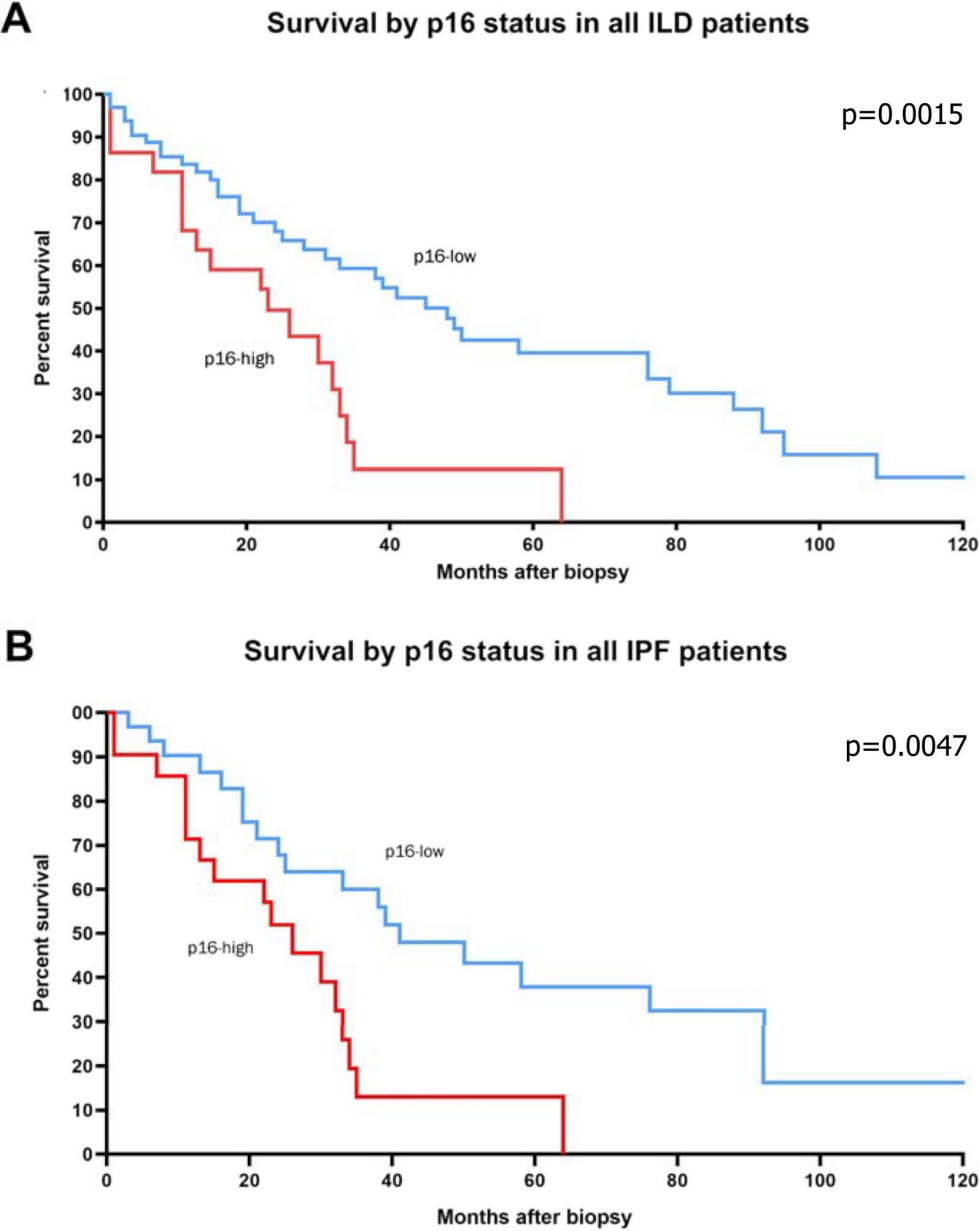
Kaplan–Meier survival analysis grouped by p16-positive foci. **A** LTx-free survival was significantly worse in all ILD patients (*p* = 0.0015) with a high density of p16-positive foci. **B** LTx-free survival was significantly worse (*p* = 0.0058) in IPF patients with a high density of p16-positive foci

LTx-free survival was significantly reduced in cases with a high density of p16-positive foci when compared to cases with a low density of senescent foci (Fig. 7A). This remained true considering IPF cases only (Fig. 7B). On regression analysis, a high level of p16 expression was indeed significantly associated with LTx or mortality (HR 2.47, C.I. 1.30–4.62, p = 0.0067). ROC analysis demonstrated that high expression of p16 (> 2.1 foci per 100 mm^2^ lung tissue) had a 33% sensitivity (95% confidence interval, 21–47%) and 87% specificity (95% confidence interval, 70–96%) in predicting post-biopsy LTx-free survival, resulting in an accuracy (area under the curve) of 60% (p = 0.025) (Additional file 3: Fig. S3).

Considering only IPF patients (and excluding 8 subjects who developed lung cancer post-biopsy), p16 remained a risk factor towards death or LTx, but antifibrotic therapy was significantly protective (HR 2.64, p = 0.0150) on multivariate analysis (Table 6). Importantly, all patients within high p16 expression who were on anti-fibrotic therapy survived (except 1 who died of cancer), while all others either died or required a LTx.

**Table 6:** Predictors of lung transplant-free survival after surgical lung biopsy in patients with IPF (after excluding patient who developed lung cancer post-biopsy)

Cox proportional hazard analysis	Hazard ratio	C.I	p value
p16 > 2.1 foci per 100 mm.^2^ of lung tissue	2.40	1.07–5.51	0.0331
Antifibrotic treatment^*^	0.28	0.10–0.66	0.0029

N = 46. Multi-variate analysis. Age, gender, BMI, FVC (% pred) and DLCO (% pred) were not significant

^*^Pirfenidone or nintedanib

## Discussion

The role of cellular senescence has become increasingly recognized as a major factor in the development of many diseases, with clinical applications for immunohistochemical markers of senescence. In this study, we were able to link immunohistochemistry for p16 to a high likehood of IPF diagnosis and also to a significantly worse outcome, when this expression was high.

Discrete foci of senescence corresponding to senescent epithelial cells and myofibroblasts were identified in recent single cell sequencing studies [14, 15]. The distribution and quantification of p16-positive foci of senescence presented as a challenge because of its low frequency and relative heterogeneity between cases. We felt that simple quantification per high powered field did not adequately standardize nor represent whether senescence appeared to be driving the underlying pathogenesis of disease. We had considered and tried to measure the number of foci in small areas (2 mm^2^ of lung tissue), but we had found this metric was too variable for these low frequency features. Therefore, we chose to normalize our analysis to the entire lung parenchyma, measuring the entire surface area of fibrosis on the slide using digital image analysis (QuPath). This assessment remains the most accurate and robust measure of the number of p16-positive foci while normalizing for differences in the amount of sampled lung tissue, opening its potential application to smaller cryobiopsy specimens for disease classification and prognostication [31]. We also confirmed the expression of other markers of senescence (p21 and pRB) on p16-positive fibroblastic foci.

One interesting finding of our study is that the presence of p16 overexpression highlights only a subset of fibroblastic foci. Some groups have suggested that the number of fibroblastic foci correlates with poor outcome [12]. However, the definition of a fibroblastic focus remains heterogeneous, with interobserver variability [13]. Therefore, this criterion alone may have limitations as a measure of disease burden. We found that using immunohistochemistry, p16 overexpression highlights a subset of fibroblastic foci, allowing for easier identification of these foci. This technique may be leveraged to clearly define these fibroblastic foci and may make this task more amenable to automation. Importantly, not all fibroblastic foci are positive for p16, suggesting that these collections of p16-positive fibroblasts are driving this disease process through cellular senescence.

We then sought to characterize the features of senescence-associated IPF. A high density of p16-positive foci was associated with a significant worse outcome, despite having no association with any specific HRCT pattern. Our data also demonstrated improved outcomes in patients with a high density of p16-positive foci that were started on these antifibrotics, serving as a marker of increased response from antifibrotic therapy. Validation of these findings in larger cohorts is necessary to determine the value of p16 in guiding management and may support a rationale to obtain the “p16 status” in biopsies for IPF.

Gene expression studies on bulk lung tissues from IPF patients have previously identified increases in extracellular matrix (ECM) remodelling genes, fibroblast proliferation and collagen deposition compared to other types of ILD and normal lung tissue [14, 32–34]. Our data builds on this work, assessing 24 different lung microenvironments from 2 IPF lung samples, using the new DSP methodology. A transcriptional increase of CDK inhibitor p21, ECM proteins and matrix metalloproteinases within the fibroblastic foci of senescence-associated IPF was observed. PCA mapping and hierarchical clustering showed a clear demarcation of fibroblastic foci from both normal areas and areas of fibrosis. These data, although only preliminary, highlight the importance of in situ tissue transcriptional analysis and support the hypothesis that fibroblastic foci play an independent role in promoting senescence-associated ECM remodelling in a UIP pattern of lung fibrosis. As a limitation, our data was based on the Cancer Transcriptome Atlas Assay, the largest available panel for FFPE tissues at the time of the study, rather than whole transcriptome. Future work to optimize FFPE and fresh-frozen tissues for transcriptome analysis may highlight pathways that were not identified with these techniques, such as the role of other ECM remodelling proteins or inhibitors that were previously classified in these original studies.

In IPF, cells reported to express senescence markers include fibroblasts and alveolar epithelial type 2 cells [16]. Single-cell RNA studies in IPF identified aberrant, cuboidal epithelial expressing multiple markers of senescence and localized at the edge of fibroblastic foci, exactly where the p16 immunostaining was observed [14]. Importantly, these cells exhibited features of epithelial-to-mesenchymal transition [14], linking senescence to a fundamental mechanism of the fibrogenic process. Animal model data indeed suggested that senescence and depletion of alveolar epithelial type 2 cells can lead to spontaneous lung fibrosis [15]. Myofibroblasts within fibroblastic foci in IPF display an apoptotic-resistant phenotype [15], a feature compatible with senescence [35]. However, a diminished proliferative capacity was observed in IPF lung myofibroblasts and is inconsistent with the proliferative process suggested in the fibroblast foci [35]. Overall, it appears that, in IPF, senescence primarily takes place in epithelial cells, but, through SASP, could have a long range of fibrogenic consequences in the microenvironment. Further studies with spatially-resolved transcriptomics on lung biopsies in treatment-naïve patients will likely help clarifying this issue and identify new targets for treatment. From a therapeutic perspective, given consistent transcriptional and immunohistochemical findings, there is an increasing interest in the use of senolytic drugs in IPF. A preliminary experience from Justice et al. with dasatinib plus quercetin demonstrated the feasibility of such trials [36].

When considering the striking difference in gene sets expression between fibroblastic foci and fibrotic areas, we should consider that the ROI “fibroblastic foci” do include the overlying epithelium. In this regard, the upregulation of the “cadherin binding” gene set in fibroblastic foci (vs. fibrotic areas) is very interesting, as it points again to their central fibrogenic role of epithelial-to-mesenchymal transition, where cadherin modulation is heavily involved [37]. Another important consideration in the interpretation of GSEA results is that “normal-looking areas” in IPF probably do not represent normal controls. A study from Luzina et al. suggested that normal-appearing areas are actually fully engaged in the disease process, with transcriptional expression of connective tissue, immune and inflammatory-related genes [38].

DSP analysis demonstrated a greater upregulation of p21 than p16 in fibroblastic foci. This is unsurprising, since recent studies have highlighted increased levels of both p16 [39] and p21 [34] in response to critically shortened telomeres, and various drivers of senescence via the p53-dependent cellular senescence pathway [18]. Other groups have been able to highlight p21-positive cells with immunofluorescence in cases of IPF [16]. While p21 appears to have a more significant difference at the gene expression level, it does not appear to translate well at the protein level, given the challenges associated with p21 outlined above. p21 is well known to be regulated at the protein level by the activity of E3 ubiquitin ligases, such as Skp1, which could explain the observed differences [40]. The expression of pRB also appears to be intact on fibroblastic foci and would provide a means for cell cycle blockade in these non-neoplastic cells. We also previously investigated p53 immunostaining, but patchy, wild-type staining was observed on the slides and p53 failed to differentiate UIP from NSIP [18].

## Conclusions

We demonstrated that digital quantification of p16-positive foci is a novel, simple, and inexpensive marker of IPF associated with cellular senescence. We were able to highlight some of the molecular pathways behind senescence-associated IPF in fibroblastic foci, identifying a family of matrix remodelling proteins and CDK inhibitors that may be involved in the rapid progression of fibrosis, leading to worse LTx-free survival. These data suggest that fibroblastic foci-specific activation of cellular senescence is a critical driver for the ongoing fibrosis in IPF. However, encouragingly, the regression analysis in IPF patients demonstrated that this driver is potentially treatable, given the protective effect of anti-fibrotic therapy, when started early post-biopsy.

In conclusion, the identification of p16-positive fibroblastic foci on lung biopsies may be diagnostically helpful and prognostically meaningful, identifying a subgroup of patients who may benefit the most from existing antifibrotic therapies, and potentially from future senolytic therapies.

## Supplementary Information

**Additional file 1.** Additional file figure legends.

**Additional file 2: Figure S1.** Sample quantification of p16-positive foci (red) within the total lung parenchyma (yellow) using QuPath.

**Additional file 3: Figure S2.** Recipient operating characteristic analysis of p16-positive foci against LTx-free survival. Area under the curve=0.599, p*=*0.025.

**Additional file 4: Figure S3.** Stratification of p16-positive foci according to the HRCT pattern (probable, indeterminate or inconsistent for UIP). No association between density of p16-positive senescent foci and HRCT pattern could be identified.

**Additional file 5: Figure S4.** Principal component analysis (PCA) across the analyzed portion of genome. Light brown: fibrotic areas; dark pink: fibroblastic foci; green: normal areas. PCA analysis shows a distinct separation between fibroblastic foci and fibrotic areas.

## Data Availability

All data generated or analysed during this study are included in this published article and its additional information files. The original datasets containing patient data that were used and analysed during the current study are available from the corresponding author on reasonable request.

## Abbreviations

CDK: Cyclin-dependent kinases
DSP: Digital spatial profiler
ECM: Extracellular matrix
FDR: False discovery ratio
FFPE: Formalin-fixed, paraffin-embedded
GSEA: Gene set enrichment analysis
HR: Hazard ratio
HRCT: High resolution chest CT scan
ILD: Interstitial lung disease
IPF: Idiopathic pulmonary fibrosis
LTx: Lung transplant
MDD: Multi-disciplinary discussion
MMP: Matrix metalloproteinase
NSIP: Nonspecific interstitial pneumonia
pRB: Purified retinoblastoma protein
PFT: Pulmonary function test
ROC: Receiver operating characteristic
ROI: Regions of interest
SASP: Senescence-associated signalling protein
SLB: Surgical lung biopsy
UIP: Usual interstitial pneumonia
