# Supplementary material for: Digital quantification of p16-positive foci in fibrotic interstitial lung disease is associated with a phenotype of idiopathic pulmonary fibrosis with reduced survival
Source: Respir Res. 2022 Jun 7;23:147. doi: 10.1186/s12931-022-02067-w (PMC9175499; doi:10.1186/s12931-022-02067-w)
Supplement: Supplementary file 1 — Additional file 1. Additional file figure legends. [file 12931_2022_2067_MOESM1_ESM.docx]

**Digital quantification of p16-positive foci in fibrotic interstitial lung disease is associated with a phenotype of idiopathic pulmonary fibrosis with reduced survival**

Jonathan Keow, Matthew J. Cecchini, Nathashi Jayawardena, Maurizio Zompatori, Mariamma G. Joseph, Marco Mura

**SUPPLEMENTAL MATERIAL**

**Supplemental Figure 1**. Sample quantification of p16-positive foci (red) within the total lung parenchyma (yellow) using QuPath.

**Supplemental Figure 2**. Recipient operating characteristic analysis of p16-positive foci against LTx-free survival. Area under the curve=0.599, p*=*0.025.

**Supplemental Figure 3**. Stratification of p16-positive foci according to the HRCT pattern (probable, indeterminate or inconsistent for UIP). No association between density of p16-positive senescent foci and HRCT pattern could be identified.

**Supplemental Figure 4**. Principal component analysis (PCA) across the analyzed portion of genome. Light brown: fibrotic areas; dark pink: fibroblastic foci; green: normal areas. PCA analysis shows a distinct separation between fibroblastic foci and fibrotic areas.
