# Supplementary figures and images for: Digital quantification of p16-positive foci in fibrotic interstitial lung disease is associated with a phenotype of idiopathic pulmonary fibrosis with reduced survival
Source: Respir Res. 2022 Jun 7;23:147. doi: 10.1186/s12931-022-02067-w (PMC9175499; doi:10.1186/s12931-022-02067-w)

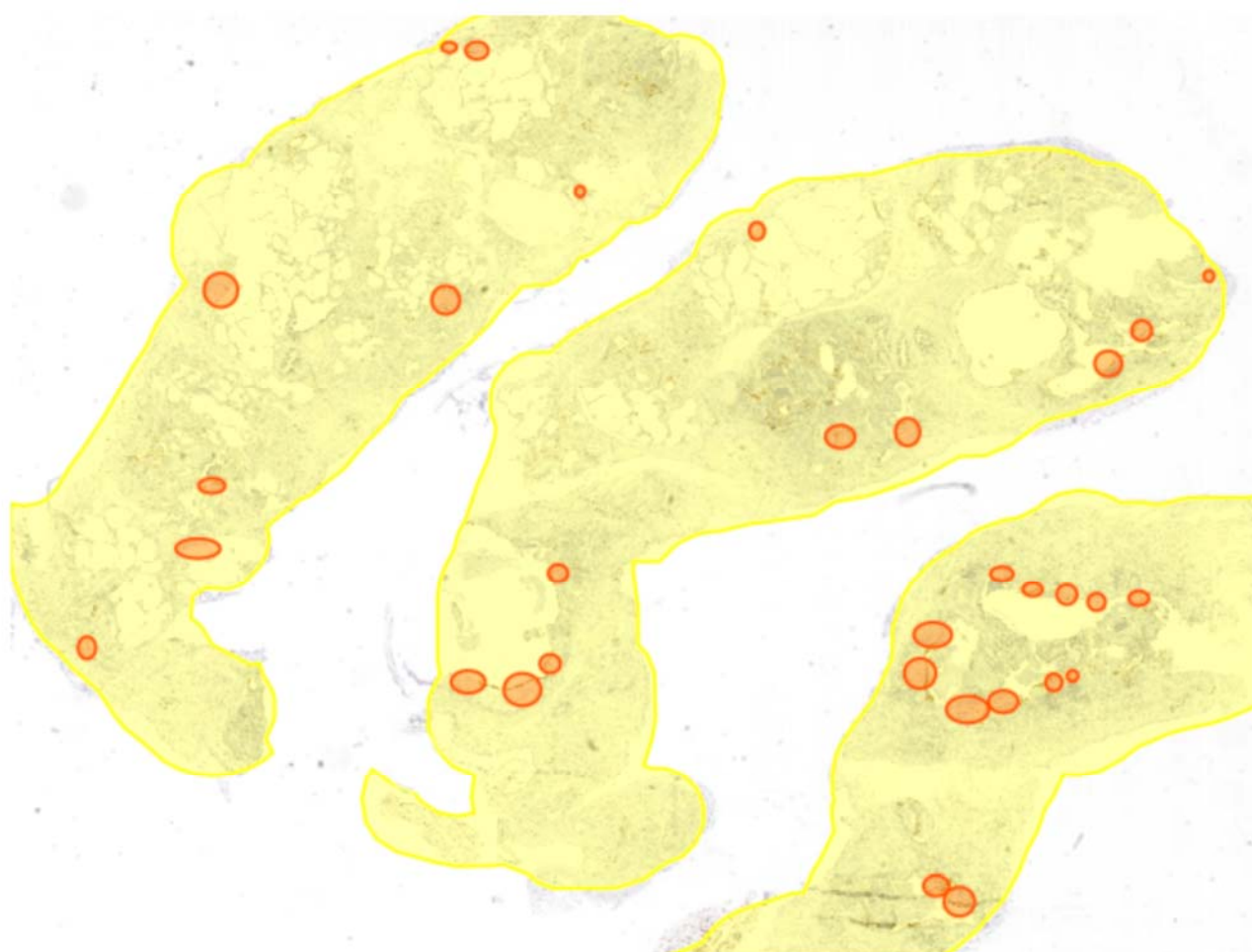

Supplement: Supplementary file 2 — Additional file 2: Figure S1. Sample quantification of p16-positive foci (red) within the total lung parenchyma (yellow) using QuPath. [file 12931_2022_2067_MOESM2_ESM.pdf]

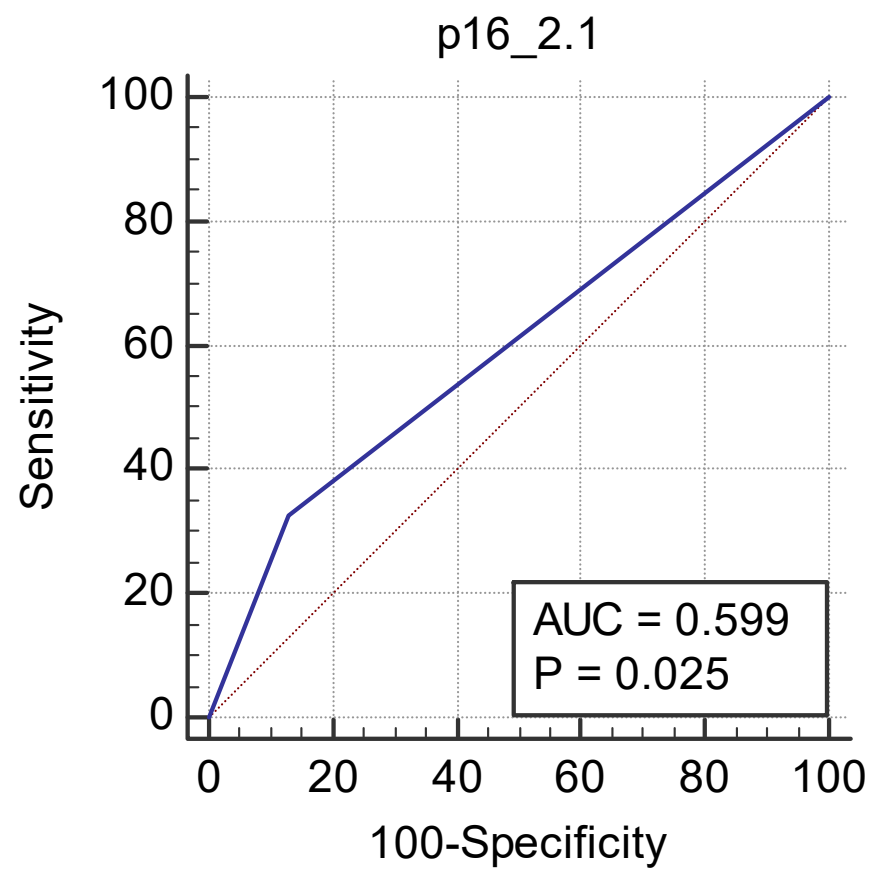

Supplement: Supplementary file 3 — Additional file 3: Figure S2. Recipient operating characteristic analysis of p16-positive foci against LTx-free survival. Area under the curve=0.599, p=0.025. [file 12931_2022_2067_MOESM3_ESM.pdf]

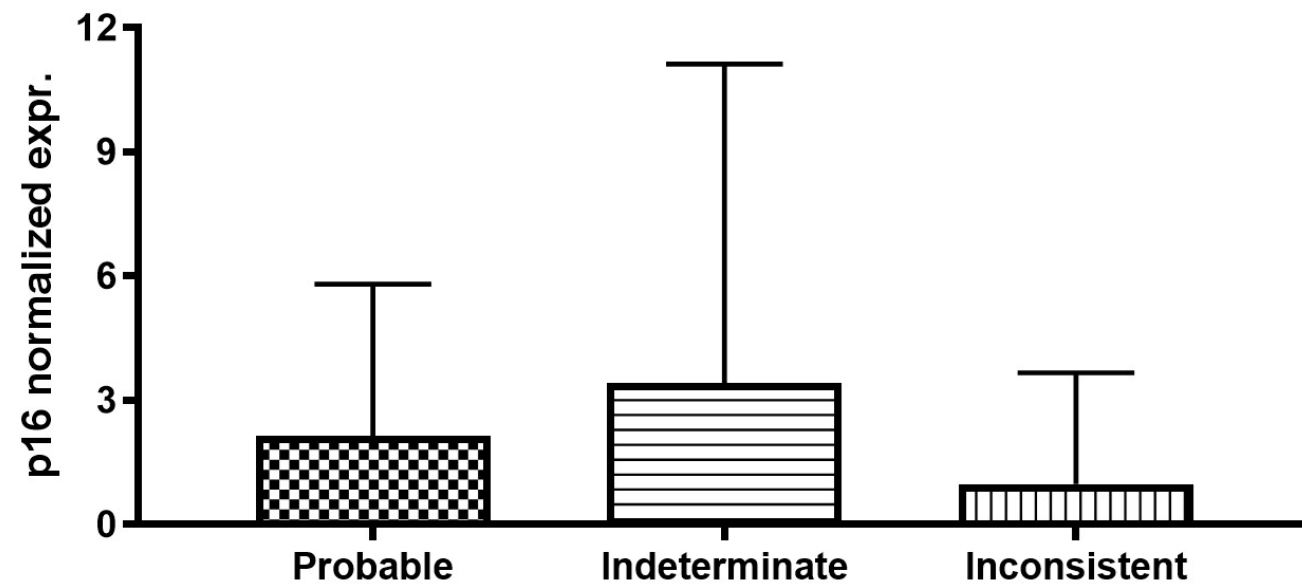

Supplement: Supplementary file 4 — Additional file 4: Figure S3. Stratification of p16-positive foci according to the HRCT pattern (probable, indeterminate or inconsistent for UIP). No association between density of p16-positive senescent foci and HRCT pattern could be identified. [file 12931_2022_2067_MOESM4_ESM.pdf]

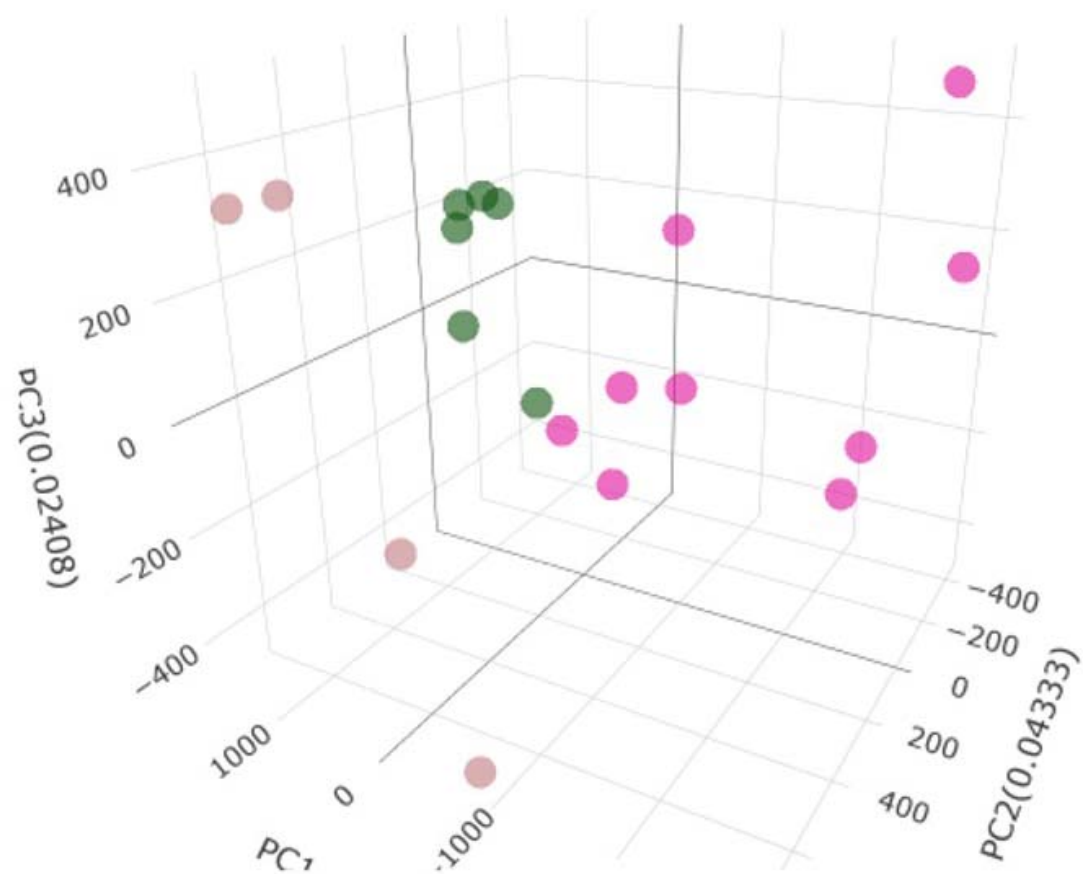

Supplement: Supplementary file 5 — Additional file 5: Figure S4. Principal component analysis (PCA) across the analyzed portion of genome. Light brown: fibrotic areas; dark pink: fibroblastic foci; green: normal areas. PCA analysis shows a distinct separation between fibroblastic foci and fibrotic areas. [file 12931_2022_2067_MOESM5_ESM.pdf]
